# Misdiagnosed appendiceal mucinous neoplasms and primary ovarian mucinous tumors present with different pre- and intraoperative characteristics

**DOI:** 10.3389/fonc.2022.966844

**Published:** 2022-08-25

**Authors:** Yi Yu, Tao Wang, Zhen Yuan, Wei Lin, Jiaxin Yang, Dongyan Cao

**Affiliations:** Department of Obstetrics and Gynecology, Peking Union Medical College Hospital, Chinese Academy of Medical Sciences & Peking Union Medical College, National Clinical Research Center for Obstetric & Gynecologic Diseases, Beijing, China

**Keywords:** misdiagnosis, appendiceal mucinous neoplasms, primary ovarian mucinous tumor, predictive factor, scoring system

## Abstract

**Objective:**

To identify the differences between the pre- and intraoperative characteristics in misdiagnosed appendiceal mucinous neoplasms (AMNs) and those in primary ovarian mucinous tumors (POMTs) and to establish an effective model for differentiating AMNs from pelvic mucinous tumors.

**Methods:**

This study enrolled 70 AMN patients who were misdiagnosed with ovarian tumors and 140 POMT patients who were treated from November 1998 to April 2021 at Peking Union Medical College Hospital. The clinical features and operative findings of the two groups of patients were collected and compared.

**Results:**

There were significant differences in age and menopausal status, but no difference in the patients’ clinical manifestations between the two groups. The preoperative serum CA125 and CA199 levels were not different between the two groups. The CEA level (31.04 ± 42.7 vs. 7.11 ± 24.2 ng/ml) was higher in the misdiagnosed AMN group (P < 0.001). The AMNs were smaller than the POMTs that were measured preoperatively by ultrasonography (US) (P<0.05) and measured at surgery (P<0.05). Furthermore, the patients with AMNs more commonly had multinodularity and ascites noted on the preoperative US (P<0.001), on CT (P<0.001), and at surgery (P< 0.001). The two groups also differed in the presence of bilateral disease, in the appendiceal appearance and peritoneal dissemination. Subsequently, a prediction model was developed using multivariable logistic regression, which was evaluated through internal validation.

**Conclusions:**

The suspicion of a nongenital organs originated tumor especially origing from appendiceal should be considered in a patient who is older, tumor size less than 12cm, multinodular, presence of mucinous ascites, and elevated serum CEA levels. Bilateral ovarian involvement, peritoneal dissemination, and an abnormal appendiceal appearance found during surgery were the typical features associated with AMNs.

## Introduction

Primary appendiceal neoplasms are rare; they represent approximately 0.5% of all cancers of the gastrointestinal tract (1) and are classified into low-grade appendiceal mucinous neoplasm (LAMN), high-grade appendiceal mucinous neoplasm (HAMN), serrated polyp, adenoma, adenocarcinoma, mucinous adenocarcinoma, and poorly differentiated adenocarcinoma with signet ring cells (2). Patients with appendiceal mucinous neoplasms (AMN) often present with nonspecific clinical manifestations, so these types of neoplasms are often not suspected before surgery. However, these tumors may be detected initially by gynecologists but can be misdiagnosed as adnexal masses due to their proximity (3). Perforated appendiceal mucinous neoplasms may cause pseudomyxoma peritonei (PMP), which is an uncommon clinical condition, and patients with PMP often present with enlarging ovarian masses and an excessive accumulation of gel-like mucinous peritoneal fluid in the peritoneal or pelvic cavity, similar to malignant ovarian tumors (4–7).

A correct preoperative diagnosis of either a primary ovarian mucinous tumor (POMT) or an AMN in female patients is very difficult due to the lack of specific tumor biomarkers and the lack of consistency in the imaging manifestations, such as in ultrasonography, computed tomography, and magnetic resonance, in these tumors (8–11). It is also difficult to distinguish between these two neoplasms intraoperatively due to the common macroscopic and microscopic features that are associated with primary and metastatic mucinous ovarian tumors (12).

It has been reported that there are a number of preoperative clinical characteristics and gross features seen during surgery that generally allow for the distinction between these two types of lesions. An algorithm using the tumor size and laterality has been evaluated and has been determined to be a relatively reliable diagnostic approach (13–15). Some other gross features, including the presence of mucinous ascites and extraovarian metastases, the surface involvement of the ovary, and a mucinous multinodular appearance, have been proven to be potential indicators of secondary tumors (16–19). Intraoperative frozen-section analysis is important to determine the correct diagnosis, but the consistency between the frozen and final pathology is partially dependent on the site, which can be difficult for gynecologists and pathologists to determine (10). Immunohistochemistry (IHC) aids in obtaining the final pathological diagnosis (20) but may not be achievable during surgery. However, a correct distinction between an AMN and a POMT both preoperatively and intraoperatively is important for avoiding an unnecessary gynecological staging surgery and insufficient exploration of the appendix (19). Therefore, this study aimed to compare the preoperative clinical characteristics and intraoperative features that can differentiate between AMNs and POMTs.

## Materials and methods

### Patient enrollment

This was a retrospective cohort study that was implemented in a tertiary teaching hospital. The Institutional Review Board from the study center approved the study. At the time of registration, all patients agreed to and signed a consent form for the collection of data obtained from their medical records and for the publication of their medical information, including relevant images. All procedures that were performed in the study and that involved human participants were performed according to the ethical standards of the institutional and/or the national research committee and the 1964 Declaration of Helsinki and its later amendments or comparable ethical standards.

A retrospective database was retrieved for review, and this database consisted of patients who were preoperatively diagnosed with pelvic masses or ovarian tumors, who initially underwent surgery performed by gynecologists and who had pathologically confirmed appendiceal mucinous neoplasms at Peking Union Medical College Hospital during the 23 years from November 1998 to April 2021. The collected information included the patient’s clinical presentation, laboratory and imaging information, surgical procedure, and pathologic information. The inclusion criteria were patients who had a mass that was located in the pelvic cavity, who had an imaging diagnosis of a gynecologic tumor before surgery (the mass was first recognized by gynecologists, and the surgical exploration was performed by gynecologists), and who had a histological diagnosis of an appendiceal mucinous neoplasm at Peking Union Medical College Hospital. Patients who had cervical or endometrial tumors and who had a correct preoperative diagnosis were excluded. The data of patients with POMT who were undergoing surgery in our department during the same period were also reviewed. The available clinical information was collected from the medical records of the patients.

### Preoperative evaluation and intraoperative findings

All of the patients in the two groups underwent evaluations before treatment, including a review of their medical history, physical examination, the measurement of tumor biomarkers, including carbohydrate antigen 125 (CA125), carcinoembryonic antigen (CEA), carcinoembryonic antigen 19-9 (CA19-9), and testing for occult blood in the stool. At least one imaging examination, including ultrasound examination (US), computed tomography (CT), magnetic resonance imaging (MRI), gastrointestinal endoscopy, or positron emission tomography-computed tomography (PET-CT), was performed before surgery, and the more detailed imaging information was evaluated, including the tumor size, consistency, modularity, calcification and the presence of ascites.

All patients were treated initially in the gynecological department and had laparoscopic or laparotomic exploration surgery that was performed by a gynecologist. The surgical records were reviewed for information regarding the type of surgery, the ovarian involvement, the tumor laterality and size, the presence of ascites, the presence of peritoneal dissemination, and the appearance of the appendix.

The pathological diagnoses that were obtained from the perioperative frozen sections were also reviewed; however, the definite diagnosis was based on the histological examination and immunohistochemistry findings from the permanent sections. The most commonly used markers were CDX2, CK20, CK7, MUC2, SATB2, PAX-8, CA125, CEA, ER, and PR (3). The AMNs were classified according to a recent consensus classification system under the auspices of the Peritoneal Surface Oncology Group International (PSOGI) (21).

### Statistical analysis

The results are presented as the mean with SD ranges for continuous data and percentages for categorical variables. We checked the normality of each variable, and most of the variables did not follow a normal distribution. Thus, we used the Mann–Whitney U rank test instead of Student’s t-test for evaluating the continuous variables and the chi-square test or Fisher’s exact test to evaluate the categorical variables. The distributions of the two populations were the same. Univariable and multivariable logistic regression analysis was performed to select predictive factors of AMNs, which formed the scoring system. The model was developed by calculating the statistical significance of combinations of factors and were validated by the chi-square values from the logistic regression and C-statistics. A P value less than 0.05 was considered significant. The data analysis was performed using Python version 3.8.5.

## Results

### Preoperative clinical characteristics

We reviewed and collected data from 70 cases of AMN that were misdiagnosed as ovarian tumors, which were then categorized into 50 patients with LAMNs and 20 patients with mucinous carcinomas, including three with signet ring cell carcinomas and one with a goblet cell carcinoid carcinoma. These patients were staged according to American Joint Committee on Cancer(AJCC) (22), four (8.0%), nine (18.0%), six (12.0%), three (6.0%) and 28 (56.0%) of the LAMNs cases were classified as confined disease, Tis, pT3, pT4aM1a and pT4bM1bG2 disease, respectively, and one (5.0%), two (10.0%), 12 (60.0%), and five (25.0%) patients with carcinomas were diagnosed with stage pT4aM1a, pT4bM1bG1, pT4aM1bG2 and pT4bM1bG3 disease, respectively.

We also included 140 control patients with POMTs, which were comprised of 96 borderline tumors and 44 carcinomas, as the comparison group. According to the International Federation of Gynecology and Obstetrics (FIGO), 74 (77.1%) and 22 (22.9%) of the POMT borderline cases were classified as stage IA and IC disease, respectively, and six (13.6%), 28 (63.6%), two (4.6%), six (13.6%) and two (4.6%) patients with carcinomas were diagnosed with stage IA, IC, II, III, and IV disease, respectively.

A comparison of the clinical characteristics of the AMN group and the POMT group is presented in **Table 1**. The median age of the patients with misdiagnosed AMNs was 58.4 (range 25-82), and 75.7% of patients were postmenopausal. Twenty-nine (41.4%) patients were admitted to the clinic for a pelvic mass or elevated tumor markers that were found during a routine physical examination. The main clinical manifestation of the patients was abdominal pain, which was reported in 13 (18.5%) patients; 14 (15.7%) patients presented with abdominal distention. The other presentations included a palpable mass or an increasing abdominal girth (12.9%) and abnormal vaginal bleeding (8.5%); the other nonspecific presentations included fever, diarrhea, and frequent urination. The median duration of the symptoms was 8.2 months (range, 0.3-48). The two groups showed significant differences in age, duration of symptoms, and menstrual status, but their clinical manifestations were similar.

**Table 1:** Preoperative characteristics of the primary ovarian mucinous tumors and misdiagnosed appendiceal mucinous neoplasms.

	Appendiceal mucinous neoplasms	Primary ovarian mucinous tumor	P value (AMN vs POMT)
n	70	140	
Pathology type	Low-grade appendiceal mucinous neoplasm (LAMN) 50/70	Borderline tumor 96/140	
	Mucinous adenocarcinoma 16/70	Mucinous adenocarcinoma 44/140	
	Mucinous adenocarcinoma with signet ring cells 3/70		
	Mucinous adenocarcinoma with goblet cell 1/70		
Age (year)	58.4 ± 14.0	39.5 ± 16.6	<0.001
Clinical presentation			
Abdominal pain	13	17	0.628
Abdominal distention	11	21	
Abdominal mass and increasing girth	9	22	
Abnormal vaginal bleeding	6	8	
Incidental finding	29	63	
Others	2	9	
Duration of presentation(month)	8.2 ± 12.9	17.4 ± 40.8	0.0295
Menstruate status			<0.001
Postmenopausal	53/70	43/140	
Premenopausal	17/70	97/140	
Tumor biomarker			
CA125(0-35.0U/ml)	67.2 ± 141.2	105.4 ± 212.0	0.126
>35.0U/ml	38/70	89/140	
normal	32/70	51/140	
CA199(0-35.0U/ml)	176.5 ± 600.0	879.1 ± 4192.8	0.1395
>35.0U/ml	21/59	46/134	
normal	38/59	88/134	
CEA(0-5.2ng/ml)	31.04 ± 42.7	7.11 ± 24.2	<0.001
>5.2ng/ml	42/60	19/117	
normal	18/60	98/117	
Occult blood in the stool			<0.001
Positive	11/34	7/26	
Negative	23/34	19/26	
Imaging examination			
Ultrasonography	65	137	
CT	43	53	
MRI	3	17	
PET-CT	9	10	
Colonoscopy	21	9	
Ultrasound	65	137	
Tumor size(cm)	10.5 ± 6.6	16.0 ± 11.3	<0.001
>12cm	17/62	69/137	
<12cm	45/62	58/137	
Consistency			0.016
cystic	32/65	63/137	
solid	5/65	1/137	
cystic-solid	28/65	73/137	
Ascites	24/65	17/137	<0.001
Nodule			<0.001
single	40/65	121/137	
multiple	25/65	16/137	
CT	43	53	
Tumor size(cm)	15.6 ± 11.1	17.4 ± 8.5	0.169
Ascites	25/43	14/53	0.003
Nodule			<0.001
single	16/43	42/53	
multiple	27/43	11/53	
Calcification	12/43	4/53	0.017
PET-CT SUVmax	2.2	4.7	
Colonoscopy			NA
Positive	0/21	0/9	
Negative	21/21	9/9	

AMN, Appendiceal mucinous neoplasms.

POMT, Primary ovarian mucinous tumor.

CA125, Carbohydrate antigen 125.

CA199, Carcinoembryonic antigen 19-9.

CEA, Carcinoembryonic antigen.

CT, Computed tomography.

MRI, Magnetic resonance imaging.

PET-CT, Positron emission tomography-computed tomography.

NA, Not applicable.

[Data was given as mean± SD or n (%)].

a: Exact tumor sizes were obtained from 62 of all 65 US reports.

The preoperative levels of the serum tumor markers are also shown in **Table 1**. The preoperative levels of CA125 and CA199 did not differ between the two groups, and the proportion of patients with elevated CA125 and CA199 levels was also similar. However, the levels of CEA and the proportion of patients with elevated CEA levels were significantly higher in the AMN group. There was a certain difference between the fecal occult blood test results between the two groups.

Of the 70 AMN patients, 65 (92.9%) patients underwent US examinations, and CT, MRI, and PET-CT were performed in 43 (61.4%), three (4.3%), and nine (12.9%) patients, respectively. Twenty-one patients underwent gastrointestinal endoscopies. **Table 2** shows the detailed results of the preoperative US and CT examinations. In the 43 (61.4%) cases who had CT performed before surgery, only two of the pelvic masses were considered as originating from the appendix. The tumor sizes that were measured in the imaging examinations of the two groups were significantly different. The AMNs tended to be smaller. There was a significant difference in the consistency of the tumors, as shown on the US. Furthermore, the presence of ascites, multinodularity, and calcification were more common in the AMN group.

**Table 2:** Surgical findings.

	Appendiceal mucinous neoplasms	Primary ovarian mucinous tumor	P value (AMN vs POMT)
n	70	140	
Ovarian involvement			<0.001
Bilateral	28	16	
Unilateral	18	124	
Uninvolved	24	0	
Size of ovarian metastasis tumor(cm)	9.2 ± 9.8 a	16.2 ± 9.1	<0.001
>12cm	23/40	88/140	
<12cm	17/40	52/140	
Appendiceal appearance			<0.001
Normal	4	139	
Abnormal	66	1	
Peritoneal dissemination			<0.001
Yes	39	8	
No	31	132	
Ascites			<0.001
Yes	38	20	
No	32	120	
Site of frozen section			
Appendix	14		
Not appendix	26		
Frozen diagnosis compared with the final diagnosis			
Appendix			
Correct	14		
Inconsistent	0		
Not appendix			
Correct	8/26		
Inconsistent	18/26		
Diagnosis during operation			
Correct	44		
Inconsistent	26		

A, data was obtained from 40 and lefted six cases presented as having surface involvement.

[Data was given as mean ± SD or n (%)].

### Intraoperative details and findings

The intraoperative findings are shown in **Table 2**. Of the 70 patients who were misdiagnosed with AMNs, 24 patients had normal ovaries or only had surface involvement noted during the operation, and these findings were confirmed to not be involved by the final pathological examinations. Seventy percent of the other patients presented with bilateral ovarian metastases, which was in contrast with the POMT group, among which only 11.4% of the patients had had bilateral disease. Consistent with previous research, the two groups had significant differences in tumor size. In 66 (94.3%) patients who had AMNs, the appendix had an abnormal appearance, and only one of the 140 patients with POMTs had an abnormal appendiceal appearance. Peritoneal dissemination and ascites were significantly more common in the AMN patients as compared to the POMT patients, and this finding is in accordance with the preoperative US and CT examinations.

We also evaluated the consistency between the fast-frozen pathological sections that were obtained during the operations and the final pathological diagnoses, which were significantly affected by the site where the frozen section was taken(C-value, 0.818).

### Predictive model and scoring system

**Figure 1:**
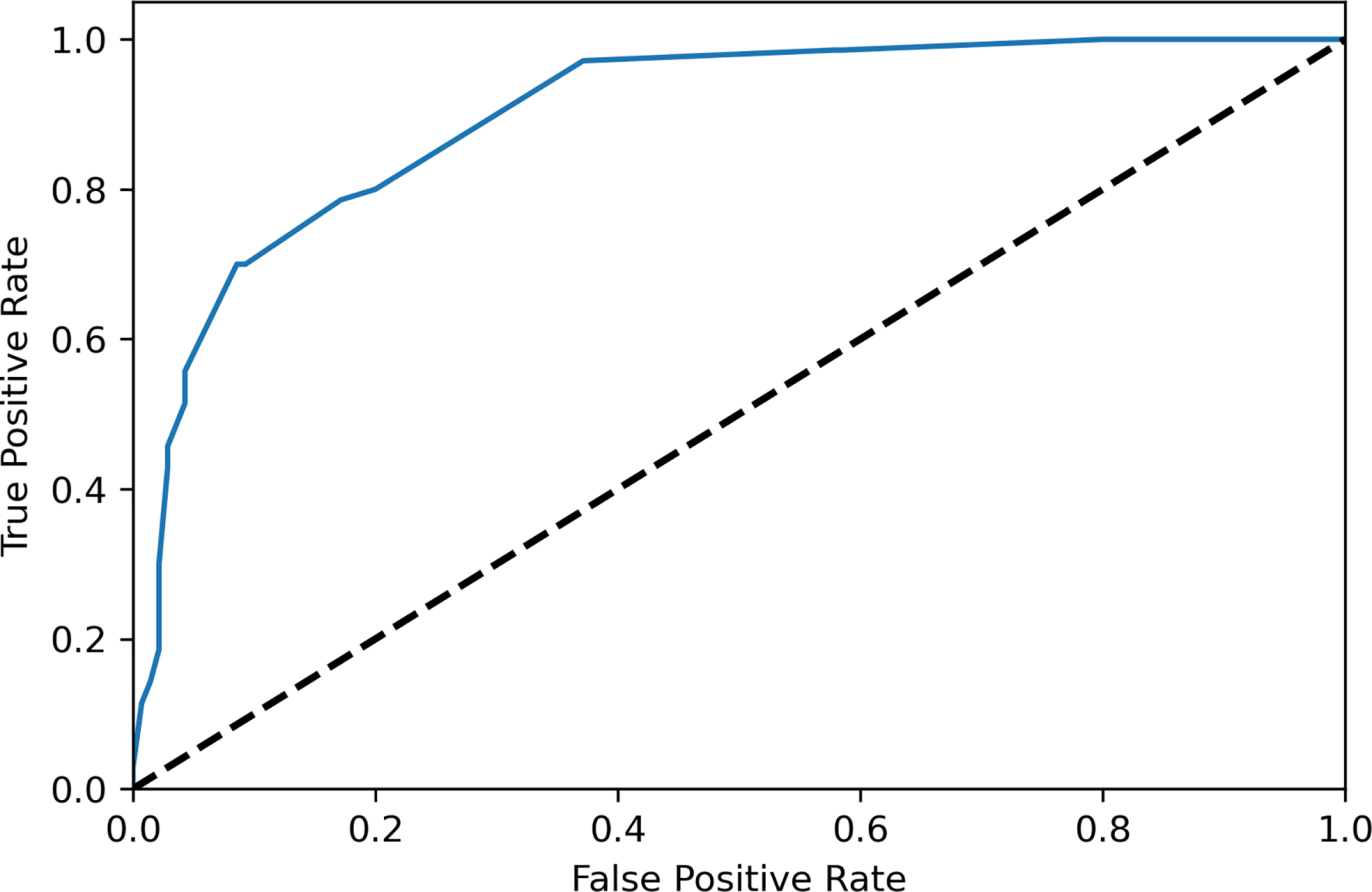
ROC curve of the scoring system was shown. The AUC was 0.899.

The variables were selected using forward variable selection based on the p value. Multivariate logistic regression analysis was performed to evaluate the predictive factors of AMNs by including age, the CEA level, the nodularity and calcification in CT, the tumor size, the laterality, and the presence of mucinous ascites (**Table 3**). Appendiceal appearance was considered a self-predictor due to its high correlation (C-value, 0.946). A scoring system was designed based on OR values of the above factors as shown in **Table 4**, which included age≥40y (aOR 7.402, 95% CI 2.688-25.003), elevated serum CEA (aOR 9.142, 95% CI 2.627-23.617), tumor size in US<12 cm (aOR 3.789, 95% CI 1.298-34.757), presence of ascites in CT (aOR 4.442, 95% CI 0.475-11.293), multiple nodularity in CT(aOR 6.603, 95% CI 0.499-11.514), calcification in CT (aOR 5.347, 95% CI 1.204-89.433), positive occult blood in the stool (aOR 2.208, 95% CI 0.265-5.432) and bilateral ovarian involvement during surgery (aOR 5.474, 95% CI 0.882-8.476). The sensitivities and specificities of different scores were shown in **Table 5** and the ROC curve of the scoring system was shown in **Figure 1**, the AUC was 0.899, which presented a good performance. The scoring system displayed the best performance during the performance analysis when the cutoff value was established at 8 points, with specificity of 0.829, sensitivity of 0.786 and accuracy of 0.814 in differentiating AMNs.

**Table 3:** The predictive factors of the AMNs were evaluated by multivariate analysis.

Logistic regression analysis				
Variables	OR	95%CI	aOR	p value
Age≥40y	8.198	2.688-25.003	7.402	0.000
CEA≥5.2ng/ml	7.878	2.627-23.617	9.142	0.000
Tumor size in US ≤12 cm	6.718	1.298-34.757	3.789	0.023
Tumor size in CT ≤12 cm	1.156	0.241-5.537	1.071	0.856
Presence of ascites in CT	2.316	0.475-11.293	4.442	0.299
Multinodularity in CT	2.397	0.499-11.514	6.603	0.275
Calcification in CT	10.377	1.204-89.433	5.347	0.033
Positive occult blood in the stool	1.201	0.265-5.432	2.208	0.813
Bilateral ovarian involvement	2.735	0.882-8.476	5.474	0.081
Intraoperative tumor size ≤12 cm	1.466	0.276-7.808	2.863	0.653

OR, Odds ratio calculated by univariate logistic regression.

CI, Confidence interval.

aOR, adjusted odds ratio calculated by multivariate logistic regression.

**Table 4:** A pre- and intraoperative clinical characteristics scoring system for differentiating AMNs from POMTs.

Variables	0	1	2	4
Age	<40y	/	/	≥40y
CEA	<5.2ng/ml	/	/	≥5.2ng/ml
Tumor size in US	≥12cm	/	<12 cm	/
Presence of ascites in CT	No	/	Yes	/
Nodularity in CT	Single	/	/	Multiple
Calcification in CT	No	/	/	Yes
Occult blood in the stool	Negative	Positive	/	/
Ovarian involvement	Unilateral	/	/	Bilateral

**Table 5:** Presentation of different cut-off scores.

Cut-off score	Estimate of possibility	Number of AMN	Total number of total patients	Rate of AMN	Sensitivity	Specificity	Accuracy
0	0.333	0	28	0.000	1.000	0.000	0.333
1	0.385	1	31	0.032	1.000	0.200	0.467
2	0.457	0	1	0.000	1.000	0.200	0.467
3	0.460	1	30	0.033	0.986	0.414	0.605
4	0.567	12	36	0.333	0.986	0.421	0.610
5	0.667	1	5	0.200	0.971	0.629	0.743
6	0.696	6	17	0.353	0.971	0.629	0.743
7	0.790	0	1	0.000	0.800	0.800	0.800
8	0.803	10	16	0.625	0.786	0.829	0.814
9	0.867	3	3	1.000	0.700	0.907	0.838
10	0.857	4	6	0.667	0.700	0.914	0.843
11	0.889	2	2	1.000	0.557	0.957	0.824
12	0.882	9	10	0.900	0.514	0.957	0.810
13	0.875	1	1	1.000	0.457	0.971	0.800
14	0.870	5	5	1.000	0.429	0.971	0.790
15	0.833	2	2	1.000	0.300	0.979	0.752
16	0.812	3	4	0.750	0.286	0.979	0.748
17	0.833	2	3	0.667	0.214	0.979	0.724
18	0.889	6	7	0.857	0.186	0.979	0.714
19	1.000	2	2	1.000	0.143	0.986	0.705
20	/	/	/	/	0.114	0.993	0.700
21	/	/	/	/	0.029	1.000	0.676

## Discussion

The present study reviewed 70 patients in the Department of Gynecology who were preoperatively misdiagnosed with ovarian tumors but were confirmed to have AMNs on final pathology and 140 diagnosed with POMTs during the same period. The clinical characteristics and intraoperative findings demonstrated that there were some significant differences in the AMN group compared with the control POMT group. However, distinguishing AMNs from ovarian tumors preoperatively is still challenging because of the nonspecific clinical manifestation and laboratory examination findings in the patients who have AMNs. AMNs could be confused with adnexal masses based on the imaging findings due to the close location, and AMNs always metastasize to ovaries (23). The surgical findings and intraoperative frozen pathology results of the appendix during surgery could give gynecologists some hints to determine the possibility of an AMN because the characteristics of AMNs can mimic the characteristics of ovarian tumors intraoperative.

Of the various demographic characteristics that were evaluated, age, duration of symptoms, and menstrual status differed between the study groups. The median age of the patients in the AMN group was 58.4 years, which is consistent with a previous study that reported that the median age at diagnosis was 59.6 years (24). Compared with AMNs and other epithelial ovarian cancers, POMT patients are generally younger at diagnosis and presentation, and the majority of patients (65–80%) are in stage I disease, which also explains the difference in the menopausal status between the two groups (25). Both tumors are often asymptomatic in the early stage due to their slow growth rate and deep location within the pelvis. Some patients have nonspecific symptoms, such as abdominal pain, distention, and increasing girth, and these symptoms are usually associated with tumor rupture, mucous accumulation from tumor implantation, or the large volume of the tumor.

Our analysis of serum tumor markers demonstrated that CA125 and CA199 are not useful in differentiating AMN and POMT because CA125 and CA199 can be elevated in patients with any source of peritoneal irritation and mucinous tumors; therefore, these markers hence lack specificity (7). A higher level of CEA can be a strong indicator of an AMN. However, CEA may also be elevated when there is a POMT (25) or other gastrointestinal (GI) tumors. Several publications have focused on the roles of CEA, CA199, and CA125 in the primary evaluation, follow-up, treatment evaluation, and prognosis prediction for patients who have appendiceal tumors (26), but few studies have focused on using these markers for diagnosis. It is still necessary for the gynecologist to consider that a pelvic mass may not originate from the adnexa when a patient has an abnormally elevated CEA level found in the preoperative examination. The fecal occult blood test and gastrointestinal endoscopy are commonly performed to exclude GI-originated tumors. Even though there was a significant difference in the results of the fecal occult blood tests between the two groups, this test did not help in determining the origin of the primary tumor due to the inaccuracy of this test. However, a positive result could help to determine the possibility of a tumor originated from gastrointestinal tract and if a patient needs a gastrointestinal endoscopy exam. None of our preoperative colonoscopies definitively diagnosed an AMN, except for three of 21 patients who were shown to have cecal ulcers on endoscopy but with no meaningful pathology. Although colonoscopy rarely yields a diagnostic biopsy (27), it is still recommended to be performed, especially given the similar histological structure between the colon and appendix (26).

The comparison of the US and CT features showed some meaningful characteristics of AMN, which tend to be a smaller size and multinodular and can present with ascites. Epstein, E. et al (28) evaluated the ADNEX model, which includes age, CA125 level, tumor solid components maximum diameter and ratio, and >10 locules and ascites, in order to differentiate between disseminated primary ovarian cancer from metastatic nonovarian cancer, and this model has a sensitivity of 82% and a specificity of 70%. In our series, 10/40 and 13/41 of the patients had a single-nodular tumor and no ascites, respectively, in the US examinations, however they were found to have the opposite situation during surgery, which indicated that US examination was not precise enough in evaluating the nodularity and the presense of ascites. In contrast, CT or MRI is more accurate in assessing mucinous tumor implantation on the peritoneum or omentum and ascites, which can present with multinodular cystic appearances and irregularly localized fluid (29), as shown in our cases. Furthermore, mural calcifications found during CT imaging were a sensitive feature for an appendix neoplasm, especially in well-differentiated LAMNs (30). There are limited data on the role of PET-CT in the diagnosis and evaluation of mucinous tumors, and PET-CT was only performed in 19 patients in the present research. There was no significant elevation in the SUV max value that was obtained from the small sample size, and this may have been caused by the fact that the majority of the patients had low-grade or borderline tumors.

The unique macroscopic appearance of AMNs, especially LAMNs, has been analyzed. Yoshida, H. et al (19) reported that a multinodular appearance, spontaneous tumor rupture, and peritoneal metastasis were frequently observed in the ovarian metastases of LAMNs but were rarely observed in POMTs. The vast majority of ovarian mucinous carcinomas are found during the early stage of the disease (FIGO I and II), whereas only 11.1% of the cases are found during the advanced stage (FIGO III and IV) (31), and the gross features of advanced stage of ovarian mucinous adenocarcinoma during surgery may also include peritoneal dissemination, omentum spread, and mucinous ascites, as well as bilateral tumors. Due to its rarity, gynecologists should always consider a diagnosis of AMN during surgery when the tumor is bilateral, is small, and has a multinodular pattern, as well as when there is ovarian involvement. The abnormal appendiceal appearance is a significant finding because of which the appendix should always be thoroughly examined during mucinous tumor operations. In the AMN patients in our study who had abnormalities in the appearances of their ovaries and appendix, the gynecologists still preferred to obtain fast-frozen biopsies from the ovaries (20/26), and 14 of the patients were misdiagnosed as having an ovarian borderline mucinous tumor or mucinous carcinoma. As indicated in our research, when the appendix was not removed for intraoperative frozen pathology, the correct diagnosis during operation was more difficult to obtain (10). In this case, abnormal appendiceal appearance was not included in the scoring system.

To the best of our knowledge, this was a large study focusing on differentiating AMNs that were misdiagnosed as primary ovarian tumors using both preoperative and intraoperative factors.The present study appears to be the first study managing to build a scoring system that has been validated to be an effective and beneficial prediction model for ensuring a practical differentiating diagnosis and proper surgical therapy. The generalizability of these results is subject to certain limitations. For instance, it was a single-center-based retrospective study, which may influence the accuracy of the multivariate analysis used to evaluate the predictors. And the long-term study period might influence the interpretation of the results, due to the changes in diagnostic tools as well as facilities over the study period as CT has been proposed by Peritoneal Surface Oncology Group International(PSOGI) as the optimal staging strategy. In spite of its limitations, this work offers a more valuable and clinically practical tool to identify misdiagnosed AMN, a rare presentation of a rare disease, for gynecological oncologists compared with previous studies. More broadly, research is also needed to be carried out on expanding the cohort to include those patients with ovarian masses who underwent primary surgery by GI surgeons for presumed preoperative AMNs. Preoperative imaging examination play an important role in our predicting scoring system, hence more research on imaging signature of AMN would help us to establish a greater degree of accuracy on this matter.

## Conclusion

The clinical manifestations of AMNs that present as pelvic masses are nonspecific and can easily be misdiagnosed as gynecological tumors, and laparoscopic exploration can be performed by gynecologists. It is important to emphasize that the complete clinical characteristics, including medical history, physical examination, different imaging methods, occult blood in stool and tumor markers can be evaluated during the preoperative evaluation. All of these factors must be evaluated with the intraoperative consultation, operative findings, and frozen tissue histopathological findings and must be combined with the gross findings (including size, laterality, nodularity, presence of mucinous ascites, peritoneal lesions, and appendiceal appearance) to achieve the best possible interpretation.

## Data availability statement

The raw data supporting the conclusions of this article will be made available by the authors, without undue reservation.

## Ethics statement

The studies involving human participants were reviewed and approved by The Institutional Review Board from Peking Union Medical College Hospital. Written informed consent to participate in this study was provided by the participants’ legal guardian/next of kin.

## Author contributions

YY: conceptualization, methodology, data curation, formal analysis, and writing - original draft. TW: data curation, investigation, and writing - review and editing. ZY: conceptualization and methodology. WL: resources and data curation. JY: project administration and writing - review and editing. DC: supervision. All authors contributed to the article and approved the submitted version.

## Conflict of interest

The authors declare that the research was conducted in the absence of any commercial or financial relationships that could be construed as a potential conflict of interest.

## Publisher’s note

All claims expressed in this article are solely those of the authors and do not necessarily represent those of their affiliated organizations, or those of the publisher, the editors and the reviewers. Any product that may be evaluated in this article, or claim that may be made by its manufacturer, is not guaranteed or endorsed by the publisher.

## References

[1] McCusker ME Cote TR Clegg LX Sobin LH. Primary malignant neoplasms of the appendix: a population-based study from the surveillance, epidemiology and end-results program, 1973-1998. Cancer (2002) 94(12):3307–12. doi: 10.1002/cncr.10589 12115365

[2] Carr NJFC Thomas D Mohamed FS Leslie H Sugarbaker PH González-Moreno S. A consensus for classification and pathologic reporting of pseudomyxoma peritonei and associated appendiceal neoplasia. Am J Surg Pathol (2016) 40(1):14–26. doi: 10.1097/PAS.0000000000000535 26492181

[3] Shaib WL Assi R Shamseddine A Alese OB Staley C. 3rd Memis B. Appendiceal mucinous neoplasms: diagnosis and management. Oncologist (2017) 22(9):1107–16. doi: 10.1634/theoncologist.2017-0081 PMC5599200 28663356

[4] Smeenk RM van Velthuysen ML Verwaal VJ Zoetmulder FA. Appendiceal neoplasms and pseudomyxoma peritonei: a population based study. Eur J Surg Oncol (2008) 34(2):196–201. doi: 10.1016/j.ejso.2007.04.002 17524597

[5] Panarelli NC YR. Mucinous neoplasms of the appendix and peritoneum. Arch Pathol Lab Med (2011) 135(10):1261–8. 10.5858/arpa.2011-0034-RA 21970481

[6] Xie X Zhou Z Song Y Li W Diao D Dang C. The management and prognostic prediction of adenocarcinoma of appendix. Sci Rep (2016) 6:39027. doi: 10.1038/srep39027 27982068 PMC5159879

[7] Mittal R Chandramohan A Moran B. Pseudomyxoma peritonei: natural history and treatment. Int J Hyperthermia (2017) 33(5):511–9. doi: 10.1080/02656736.2017.1310938 28540829

[8] Papoutsis D Protopappas A Belitsos P Sotiropoulou M Antonakou A Loutradis D. Mucocele of the vermiform appendix misdiagnosed as an adnexal mass on transvaginal sonography. J Clin Ultrasound (2012) 40(8):522–5. doi: 10.1002/jcu.20858 21739436

[9] Tanaka YO Takazawa Y Matsuura M Omatsu K Takeshima N Matsueda K. MR imaging of secondary massive ovarian edema caused by ovarian metastasis from appendiceal adenocarcinoma. Magn Reson Med Sci (2019) 18(2):111–2. doi: 10.2463/mrms.ci.2017-0146 PMC6460129 29515086

[10] Zhang W Tan C Xu M Wu X. Appendiceal mucinous neoplasm mimics ovarian tumors: Challenges for preoperative and intraoperative diagnosis and clinical implication. Eur J Surg Oncol (2019) 45(11):2120–5. doi: 10.1016/j.ejso.2019.08.004 31462390

[11] Yu XR Mao J Tang W Meng XY Tian Y Du ZL. Low-grade appendiceal mucinous neoplasms confined to the appendix: clinical manifestations and CT findings. J Investig Med (2020) 68(1):75–81. doi: 10.1136/jim-2018-000975 PMC6996116 31300469

[12] Dundr P Singh N Nozickova B Nemejcova K Bartu M Struzinska I. Primary mucinous ovarian tumors vs. ovarian metastases from gastrointestinal tract, pancreas and biliary tree: a review of current problematics. Diagn Pathol (2021) 16(1):20. 33706757 10.1186/s13000-021-01079-2 PMC7953678

[13] Jeffrey D Seidman MD Robert J Kurman MD Brigitte M Ronnett MD. Primary and metastatic mucinous adenocarcinomas in the ovaries. Am J Surg Pathol (2003) 27(7):985–93. 10.1097/00000478-200307000-00014 12826891

[14] Khunamornpong S Suprasert P Pojchamarnwiputh S Na Chiangmai W Settakorn J Siriaunkgul S. Primary and metastatic mucinous adenocarcinomas of the ovary: Evaluation of the diagnostic approach using tumor size and laterality. Gynecol Oncol (2006) 101(1):152–7. doi: 10.1016/j.ygyno.2005.10.008 16300822

[15] Yemelyanova AVV Judson R Wu K Lee-Shu-Fune; R Brigitte M. Distinction of primary and metastatic mucinous tumors involving the ovary: analysis of size and laterality data by primary site with reevaluation of an algorithm for tumor classification. Am J Surg Pathol (2008) 32(1):128–38. doi: 10.1097/PAS.0b013e3180690d2d 18162780

[16] Antila R Jalkanen J Heikinheimo O. Comparison of secondary and primary ovarian malignancies reveals differences in their pre- and perioperative characteristics. Gynecol Oncol (2006) 101(1):97–101. doi: 10.1016/j.ygyno.2005.09.046 16278010

[17] Leen SL Singh N. Pathology of primary and metastatic mucinous ovarian neoplasms. J Clin Pathol (2012) 65(7):591–5. doi: 10.1136/jclinpath-2011-200162 22075188

[18] Stewart CJ Ardakani NM Doherty DA Young RH. An evaluation of the morphologic features of low-grade mucinous neoplasms of the appendix metastatic in the ovary, and comparison with primary ovarian mucinous tumors. Int J Gynecol Pathol (2014) 33(1):1–10. doi: 10.1097/PGP.0b013e318284e070 24300528

[19] Yoshida H Tanaka H Tsukada T Abeto N Kobayashi-Kato M Tanase Y. Gross mucinous multinodular appearance aids in the identification of ovarian metastases in low-grade appendiceal mucinous neoplasms during intraoperative consultation. Ann Diagn Pathol (2021) 50:151641. doi: 10.1016/j.anndiagpath.2020.151641 33189966

[20] Kubecek O Laco J Spacek J Petera J Kopecky J Kubeckova A. The pathogenesis, diagnosis, and management of metastatic tumors to the ovary: a comprehensive review. Clin Exp Metastasis (2017) 34(5):295–307. doi: 10.1007/s10585-017-9856-8 28730323 PMC5561159

[21] Carr NJ Bibeau F Bradley RF Dartigues P Feakins RM Geisinger KR. The histopathological classification, diagnosis and differential diagnosis of mucinous appendiceal neoplasms, appendiceal adenocarcinomas and pseudomyxoma peritonei. Histopathology (2017) 71(6):847–58. 10.1111/his.13324 28746986

[22] Martin-Roman L Lozano P Vasquez W Palencia N Gomez Y Fernandez-Acenero MJ. Defining stage in mucinous tumours of the appendix with peritoneal dissemination: the importance of grading terminology: systematic review. BJS Open (2021) 5(4):zrab059. 10.1093/bjsopen/zrab059 PMC8342933 34355239

[23] Xiao J Li P Liu W. Analysis of clinical characteristics of low-grade appendiceal mucinous neoplasm (lamn): a retrospective cohort study of 51 lamn patients. J Invest Surg (2021) 34(7):721–7. 10.1080/08941939.2019.1695986 31906733

[24] Shaib WL Goodman M Chen Z Kim S Brutcher E Bekaii-Saab T. Incidence and survival of appendiceal mucinous neoplasms: a seer analysis. Am J Clin Oncol (2017) 40(6):569–73. 10.1097/COC.0000000000000210 26270447

[25] David M Gershenson AO Ray-Coquard I. Management of rare ovarian cancer histologies. J Clin Oncol (2019) 37(27):2406–15. 10.1200/JCO.18.02419 31403866

[26] Govaerts K Lurvink RJ De Hingh Ihjt Van der Speeten K Villeneuve L Kusamura S. Appendiceal tumours and pseudomyxoma peritonei: Literature review with PSOGI/EURACAN clinical practice guidelines for diagnosis and treatment. Eur J Surg Oncol (2021) 47(1):11–35. doi: 10.1016/j.ejso.2020.02.012 32199769

[27] Trivedi AN Levine EA Mishra G. Adenocarcinoma of the appendix is rarely detected by colonoscopy. J Gastrointest Surg (2009) 13(4):668–75. doi: 10.1007/s11605-008-0774-6 19089515

[28] Epstein E Van Calster B Timmerman D Nikman S. Subjective ultrasound assessment, the ADNEX model and ultrasound-guided tru-cut biopsy to differentiate disseminated primary ovarian cancer from metastatic non-ovarian cancer. Ultrasound Obstet Gynecol (2016) 47(1):110–6. doi: 10.1002/uog.14892 25925783

[29] Koyama T Mikami Y Saga T Tamai K Togashi K. Secondary ovarian tumors: spectrum of CT and MR features with pathologic correlation. Abdom Imaging (2007) 32(6):784–95. doi: 10.1007/s00261-007-9186-4 17318680

[30] Monsonis B Zins M Orliac C Mandoul C Boulay-Coletta I Curros-Doyon F. Retrospective case-control study to predict a potential underlying appendiceal tumor in an acute appendicitis context based on a CT-scoring system. Eur J Radiol (2021) 136:109525. doi: 10.1016/j.ejrad.2021.109525 33454458

[31] Perren TJ. Mucinous epithelial ovarian carcinoma. Ann Oncol (2016) 27 Suppl 1:i53–7. doi: 10.1093/annonc/mdw087 27141073

